# Effects of interferential current electrical stimulation (IFCS) on mastication and swallowing function in healthy young adults: A preliminary study

**DOI:** 10.1002/cre2.748

**Published:** 2023-05-09

**Authors:** Yoshiki Iizumi, Yoshiaki Ihara, Joji Koike, Koji Takahashi

**Affiliations:** ^1^ Department of Special Needs Dentistry, Division of Oral Functional Rehabilitation Medicine, School of Dentistry Showa University Tokyo Japan

**Keywords:** deglutition, electrical stimulation therapy, mastication

## Abstract

**Objectives:**

This study aimed to investigate the effects of interferential current electrical stimulation (IFCS) on masticatory and swallowing function.

**Materials and Methods:**

Twenty healthy young adults were enrolled. The measurement items were spontaneous swallowing frequency (SSF), voluntary swallowing frequency (VSF), saliva secretion volume (SSV), glucose elution volume (GEV), and velocity of chew (VOC). All participants underwent both IFCS and sham stimulation (without stimulation, sham). Two sets of independent IFCS electrodes were placed on the bilateral neck. The precise location of the upper electrodes was just below the angle of the mandible, while the lower electrodes were placed at the anterior border of the sternocleidomastoid muscle. The intensity of IFCS was determined to be one level below the perceptual threshold that all participants felt discomfort. Statistical analysis was performed using a two‐way repeated measures analysis of variance.

**Results:**

For IFCS, the results of each measurement before and during stimulation were SSF: 1.16 and 1.46, VSF: 8.05 and 8.45, SSV: 5.33 and 5.56 g, GEV: 171.75 and 208.60 mg/dL, and VOC: 87.20 and 95.20, respectively. SSF, GEV, and VOC during stimulation were significantly increased by IFCS (SSF, *p* = .009; GEV, *p* = .048; and VOC, *p* = .007). Following sham stimulation, the results were SSF: 1.24 and 1.34, VSF: 7.75 and 7.90, SSV: 5.65 and 6.04 g, GEV: 176.45 and 187.35 mg/dL, and VOC: 91.35 and 88.25, respectively.

**Conclusion:**

While no significant differences were observed in the sham group, our findings suggest that IFCS of the superior laryngeal nerve may impact not only the swallowing function but also the masticatory function.

## 1 INTRODUCTION

Various diseases, such as cerebrovascular disease, neuromuscular disease, dementia, and head and neck cancer have been reported to cause dysphagia (Clarke et al., 1998; Cohen et al., 2016; Niimi et al., 2018; Pflug et al., 2018; Takizawa et al., 2016). Japan is a super‐aging society, (United Nations, 2022) where a significant number of individuals live with dysphagia as a result of aging, without any apparent underlying disease. This poses a major problem in the medical care of the elderly population. Rehabilitation methods for managing dysphagia are classified as either direct or indirect training. Because most indirect training methods require patient compliance, patients with feeding and swallowing difficulties may face limitations in following the training instructions. Therefore, direct training is often performed using compensatory methods such as postural techniques and modifying the amount or consistency of bolus material (Nohara, 2020).

Direct rehabilitation strategies have been reported to induce a positive effect on the transfer of food from the oral cavity to the pharynx, a decrease in residue in the pharynx, and a decrease in the incidence of aspiration pneumonia during mealtimes (Alghadir et al., 2017; Ertekin, 2011; Groher, 1987; Tachimura et al., 2005) and involved active exercises, often rigorous, during the specific task of swallowing; “sensorimotor with swallowing” (Rogus‐Pulia & Robbins, 2013). In contrast, indirect strategies include stimulation of the oral and pharyngeal structures without the specific task of swallowing or “sensorimotor without swallowing” exercises to improve the range of motion and strengthen swallowing musculature (Rogus‐Pulia & Robbins, 2013). Moreover, it has been reported that electrical stimulation has the potential to increase the swallowing function in dysphagic patients who could not follow training instructions (Alamer et al., 2020; Barikroo & Clark, 2022). Electrical stimulation is widely applied in dysphagia treatment to improve suprahyoid muscle function.

Two types of electrical stimulations have been reported for the treatment of dysphagia: neuromuscular electrical stimulation (NMES), which directly affects the target muscle or muscle group (Maeda et al., 2017), and interferential current electrical stimulation (IFCS), which stimulates the nerves associated with swallowing (Carnaby‐Mann & Crary, 2007).

NMES has been used clinically to induce muscle contraction by direct electrical stimulation of muscles (e.g., suprahyoid muscle group), in patients with dysphagia. Additionally, it has been reported to be effective in increasing muscle strength and preventing muscle atrophy in the target muscle group, with most NMES applied at 0–25 mA current stimulation, within the low‐frequency range (10–100 Hz) (Heijnen et al., 2012; Ludlow et al., 2007; Park et al., 2018; Tang et al., 2017; Toyama et al., 2014; Zhang et al., 2016). In contrast, IFCS was developed to decrease the threshold of the swallowing reflex by stimulating the target sensory nerve from the body surface at the sensory threshold level. Studies have shown that in normal participants, IFCS performed at a 50‐Hz beat frequency, consisting of 2000 and 2050 Hz carrier frequencies, was most effective in promoting the swallowing reflex that does not involve muscle contraction in the case of sensory threshold stimulation in swallowing exercises for normal participants (Furuta et al., 2012). Moreover, it has been reported that IFCS improves pharyngeal swallowing function as an immediate effect in patients with dysphagia secondary to stroke or Parkinson's disease (Sugishita et al., 2018). In addition, the IFCS application is independent of the patient's ability to follow instructions.

A recent study reported that IFCS applied at a sensory threshold level could reduce the risk of aspiration in patients with dysphagia (Hara et al., 2020). Animal studies on guinea pigs have demonstrated that applying IFCS to the superior laryngeal nerve can reduce the latency period for swallowing (Umezaki et al., 2018). However, the details of its effect on masticatory and swallowing functions remain unclear. Furthermore, no study has investigated the effects of IFCS on the sequence of the oral and pharyngeal aspects of swallowing; a relationship between IFCS and masticatory behavior has also been previously suggested (Iizumi et al., 2022). This study aimed to clarify the effect of IFCS on mastication and swallowing function in healthy adult participants.

## 2 METHODS

### 2.1 Participants

A total of 20 healthy young adults aged 20–40 years (9 males, 11 females; mean age, 27.40 years; standard deviation, SD = 3.39) were included as participants in this study. The subjects were interviewed orally, and their medical history was investigated before participation in this study. Exclusion criteria were as follows: (1) difficulty in mastication or swallowing; (2) medical history that might affect mastication and swallowing function; (3) taking medication that affects mastication and swallowing function; (4) symptoms that might affect mastication and swallowing function, such as the sore throat or toothache on the day of measurement; and (5) use of a pacemaker.

This study was approved by the local Institutional Review Board (approval no. DH20200182) and was conducted in accordance with the World Medical Association Declaration of Helsinki (version 2002). Before enrollment, all participants were informed about the study orally and in writing and signed an approved written informed consent form.

### 2.2 Measurement conditions

To avoid the influence of circadian variation and measurement environments, such as time and room temperature, all participants were measured in the same environment and at the same time of the day. The participants were instructed not to eat or drink for 1 h before evaluation and to not speak during the measurement period. During the measurements, the participants were seated in a chair in a resting posture. The measurements were conducted over 2 days, with a 1‐week interval between the first and second measurements.

### 2.3 Electrical stimulation

**Figure 1:**
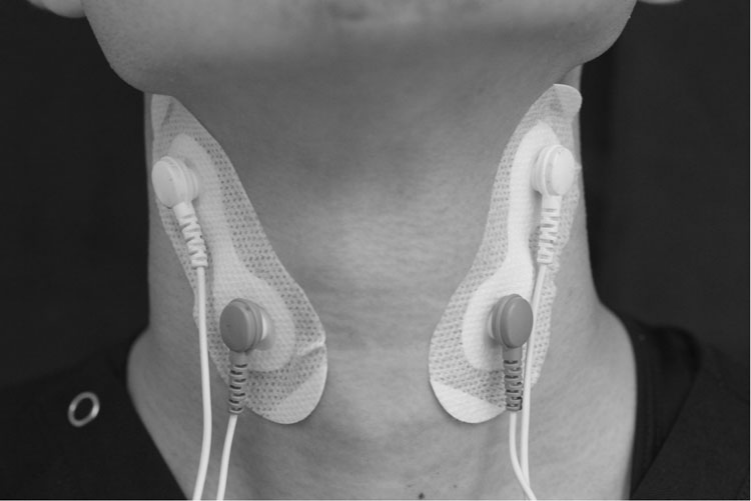
GENTLE STIM® and electrode settings. The electrodes are attached to both sides of the neck; the upper part is located just below the angle of the mandible, and the lower part is located at the anterior border of the sternocleidomastoid muscle. The IFCS is delivered by cross‐current electrical stimulation with a beat frequency of 50 Hz generated by a carrier frequency of 2000 and 2050 Hz electrical stimulation. IFCS, interferential current electrical stimulation.

In this study, IFCS was performed using “GENTLE STIM” (Food Care Co.). In the control group (sham stimulation; Sham), the wires inside the GENTLE STIM device were disconnected. Therefore, although the device was identical to the one used for the IFCS, no stimulation was transmitted to the participants in the control group (Sham). The method used to determine the sensory threshold involved incrementally increasing the intensity of the stimulation to the participant until the minimum threshold of perception was reached. For the purpose of the participants who did not feel any discomfort and were blinded as possible to the Sham, the stimulus intensity used in this study was set at 0.2 mA below the level at which participants reported a feeling of discomfort. While the sensory threshold for IFC in healthy adults is typically reported to be 1 mA (Oku, 2021), the average stimulus intensity used in this study among 20 subjects was averaged at approximately 1.6 mA (SD = 0.33). Two sets of independent IFCS electrodes were placed across the participants' thyroid cartilage to stimulate the superior laryngeal nerve. The precise location of the upper electrodes was just below the angle of the mandible, and the lower electrodes were placed at the anterior border of the sternocleidomastoid muscle (Figure 1).

### 2.4 Measurement items

During the evaluation, the following five measurements were performed before and during stimulation on Days 1 and 2, respectively.

#### 2.4.1 Spontaneous swallowing frequency (SSF)

During measurements, the participants were instructed to sit in a resting position without performing any actions that would affect salivation, such as vocalization or unnecessary jaw movements. The number of spontaneous swallows was measured over a 10‐min period, and the SSF per minute was calculated as the SSF of the participant. The number of spontaneous swallows was measured by counting the swallowing sounds using a microphone placed over the skin lateral to the trachea, just below the cricoid cartilage (Takahashi et al., 1994). Each participant also counted the number of swallows using a manual counter. If there was a difference in the number of spontaneous swallows between the two methods, the result obtained from the swallowing sounds method was selected as the participant's measurement. The frequency of spontaneous swallowing is a significant indicator of dysphagia severity in various neurologic populations (Crary et al., 2022).

#### 2.4.2 Voluntary swallowing frequency (VSF)

VSF was measured by administering the 30‐s repetitive saliva swallowing test (RSST). The measurement the voluntary swallowing in 30 s has been reported to have a sensitivity and specificity of 0.98 and 0.66, respectively. If the results are more than three empty swallows in 30 s, the possibility of aspiration is very low (Oguchi et al., 2000).

#### 2.4.3 Saliva secretion volume (SSV)

SSV was measured using the Saxon test. The participants chewed on sterile gauze for 2 min. The volume of saliva produced was calculated from the change in the gauze weight before and after chewing. A gauze weight of 2 g or less in 2 min is considered to indicate the presence of xerostomia.

#### 2.4.4 Masticatory ability (glucose elution volume, GEV)

**Figure 2:**
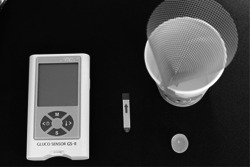
GLUCOSENSOR® is used for mastication assessment. Participants freely chewed the gummy jelly (GLUCOLAM®) for 20 s, took 10 mL water into their mouth without swallowing, and spit out all the water, saliva, and GLUCOLAM® together into the disposable filtration mesh to collect the filtrate. The collected filtrate was dotted on the sensor tip of the GLUCOSENSOR GS‐II G.C®, and the glucose elution volume was measured.

Masticatory ability was measured by glucose elution volume using gummy jelly for masticatory performance assessment (GLUCOLAM; GC Corporation). The participants were instructed to chew habitually for 20 s. Subsequently, 10 mL of water was poured into the oral cavity and the participant was asked to spit it out into a cup with a filter, together with the masticated gummy jelly, saliva, and water. The spit‐out water was then collected using a micro brush, and the amount of eluted glucose was measured using a glucose concentration tester (GLUCOSENSOR GS‐II; GC Corporation) as a measure of masticatory ability (Figure 2). The measurements are expressed as a glucose concentration; a value of less than 100 mg/dL is considered to indicate reduced masticatory capacity (Kobayashi et al., 2006; Minakuchi et al., 2016).

#### 2.4.5 Velocity of chew (VOC)

**Figure 3:**
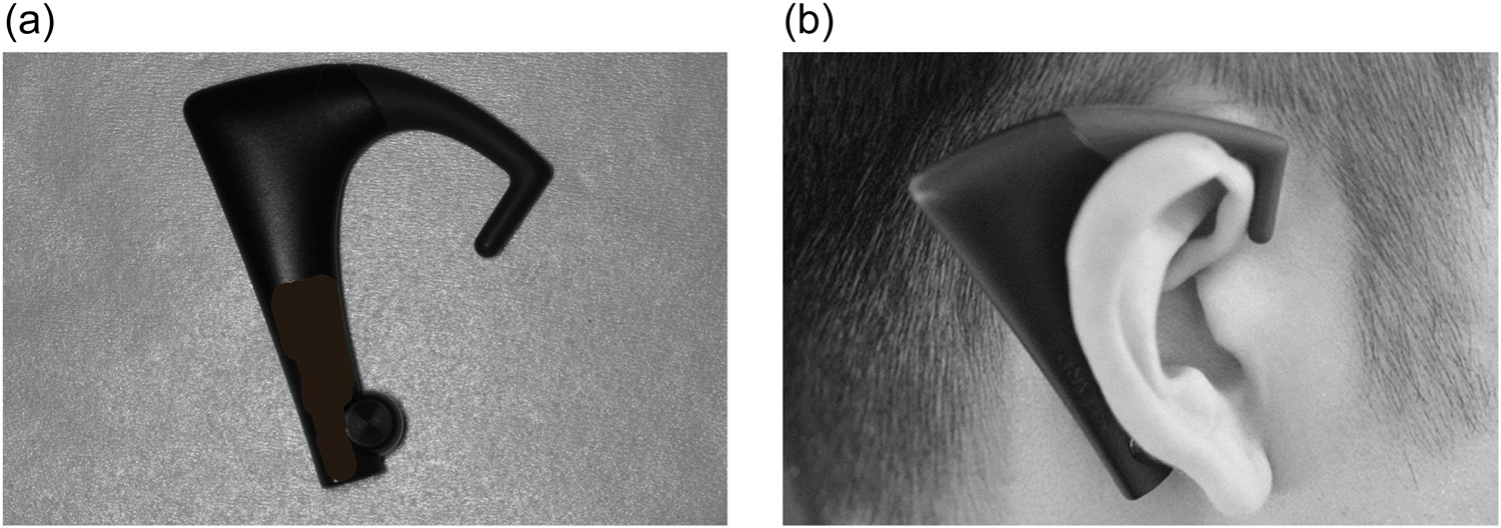
Description of Bitescan®. (a) The appearance of BITESCAN®. (b) BITESCAN® can measure the frequency of mastications, mastication time, and changing posture during mealtime by scanning the changes in the skin shape of the auricular lining.

The number and VOCs were measured using a wearable device (BITESCAN; Sharp Corporation) (Figure 3) during the measurement of masticatory ability. We used an infrared distance sensor: a device that measures the changes in the skin shape at the back of the auricular region caused by the forward movement of the articular head of the mandible during mastication; furthermore, it measures the frequency of mastication and mastication speeds by continuously scanning the changes in skin shape (Hori, Uehara, Yoshimura, et al., 2021). The accuracy, precision, and recall of this device are 101.6 ± 13.6%, 85.3 ± 11.0%, and 84.5 ± 9.5%, respectively (Hori, Uehara, Yamaga, et al., 2021).

### 2.5 Procedure

**Figure 4:**
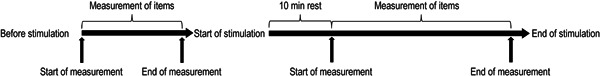
Measurement procedure. Five items (SSF, VSF, SSV, GEV, and VOC) were measured before stimulation. The stimulation was started and applied for 10 min, and then the five items were measured again during stimulation. One week later, the stimulus application for each participant was reversed compared to the one applied on Day 1 and the same procedure was used for the measurements. GEV, glucose elution volume; SSF, spontaneous swallowing frequency; SSV, saliva secretion volume; VOC, velocity of chew; VSF, voluntary swallowing frequency.

On the first measurement day (Day 1), the five items described above were measured before the application of stimulation. Thereafter, all participants randomly received either IFCS or sham. After 10 min of stimulation, the five items were measured again during the application of stimulation. After a 1‐week interval, on the second measurement day (Day 2), the five items were measured before the application of stimulation. This time, the type of stimulation applied to all participants was reversed from the one they received on the first day. After stimulus application for 10 min, the five items were measured again during the application of stimulation (Figure 4). The study was a single‐blind, randomized controlled trial in which the participants were unaware of which stimulus was used on both days.

### 2.6 Sample size

The requisite sample size was calculated using JMP ver 16.0 (SAS Institute Inc.). As the required effect size of the SSF following IFCS in healthy young individuals was reportedly 0.50 (Furuta et al., 2012), the effect size, *α* value, and detection power in the present study were determined to be 0.50, .05, and 0.8, respectively. The minimum number of participants required for the study was determined to be 19. The participants in the present study were healthy young individuals. Thus, based on a predicted withdrawal rate of 0%, we made the informed decision to enroll at least 20 participants.

### 2.7 Statistical analysis

The Shapiro–Wilk test was used to assess data normality. Measurements in both the IFCS and Sham conditions were compared before and during stimulation, and statistical analysis was performed using a two‐way repeated measures analysis of variance. The significance level for this study was set at <5%. Statistical analyses were performed using the statistical analysis software SPSS for Windows (IBM SPSS Statistics ver. 26; IBM Co.).

## 3 RESULTS

### 3.1 SSF

The results of SSF are presented in Table 1. The SSF following IFCS significantly increased from 1.16/m (SD = 0.47) before stimulation to 1.46/m (SD = 0.68) during stimulation (*p* = .009). Conversely, the SSF for Sham did not indicate a significant difference between before stimulation (1.24/m, SD = 0.24) and during stimulation (1.34/m, SD = 0.50) (*p* = .496). Furthermore, no significant difference was observed between each baseline (before stimulation) (IFCS before stimulation vs. Sham before stimulation: *p* = .618).

**Table 1:** Results of measurements.

	IFCS (*n* = 20)			Sham (*n* = 20)		
	Before stimulation	During stimulation	*p* Value	Before stimulation	During stimulation	*p* Value
SSF	1.16 (0.47)	1.46 (0.68)	.009**	1.24 (0.24)	1.34 (0.50)	.496
VSF	8.05 (2.09)	8.45 (1.61)	.501	7.75 (1.94)	7.90 (1.83)	.803
SSV	5.33 (1.96)	5.56 (2.24)	.726	5.65 (2.72)	6.04 (2.72)	.621
GEV	171.75 (54.55)	208.6 (71.71)	.048*	176.45 (63.92)	187.35 (65.07)	.596
VOC	87.2 (12.85)	95.20 (16.98)	.007**	91.35 (21.44)	88.25 (26.23)	.684

*Note*: Data presented as mean ± standard deviation or *n* (%). There are no significant differences between measurements for Sham. SSF, GEV, and VOC during stimulation for IFCS were significantly increased compared to those before stimulation (SSF, *p* = .009; GEV, *p* = .048; and VOC, *p* = .007).

Abbreviations: GEV, glucose elution volume; IFCS, interferential current electrical stimulation; SSF, spontaneous swallowing frequency; SSV, saliva secretion volume; VOC, velocity of chew; VSF, voluntary swallowing frequency.

* *p* < .05

** *p* < .01.

### 3.2 VSF

The results of VSF are presented in Table 1. The VSF was measured before (8.05/30 s, SD = 2.09) and during (8.45/30 s, SD = 1.61) IFCS stimulation. In the VSF following sham, the mean frequency was 7.75 times/30 s (SD = 1.94) and 7.90 times/30 s (SD = 1.83) before and during stimulation, respectively (Table 1). The VSF results did not reveal a significant difference among all conditions (IFCS before stimulation vs. IFCS during stimulation: *p* = .501; Sham before stimulation vs. Sham during stimulation: *p* = .803; IFCS before stimulation vs. Sham before stimulation: *p* = .641).

### 3.3 SSV

The results of SSV are presented in Table 1. The SSV was measured before (5.33 g/2 m, SD = 1.96) and during (5.56 g/2 m, SD = 2.24) IFCS stimulation. The SSV following Sham before and during stimulation were 5.65 g/2 m (SD = 2.72) and 6.04 g/2 m (SD = 2.72), respectively. The results of SSV did not indicate a significant difference among all conditions (IFCS before stimulation vs. IFCS during stimulation: *p* = .726; Sham before stimulation vs. Sham during stimulation: *p* = .621; IFCS before stimulation vs. Sham before stimulation: *p* = .632).

### 3.4 GEV

The results of GEV are presented in Table 1. The GEV following IFCS significantly increased from 171.75 mg/dL (SD = 54.55) before stimulation to 208.60 mg/dL (SD = 71.71) during stimulation (*p* = .048). In contrast, Sham showed a mean of 176.45 mg/dL (SD = 63.92) before stimulation and 187.35 mg/dL (SD = 65.07) during stimulation, with no significant difference (*p* = .596). Moreover, no significant difference was observed between baselines (before stimulation) (IFCS before stimulation vs. Sham before stimulation: *p* = .462).

### 3.5 VOC

The results of GEV are presented in Table 1. The VOC following IFCS significantly increased from 87.20/m (SD = 12.85) before stimulation to 95.20/m (SD = 16.98) during stimulation (*p* = .007). In contrast, the VOC following Sham did not indicate a significant difference between before stimulation (91.35/m, SD = 21.44) and during stimulation (88.25/m, SD = 26.23) (*p* = .684). Moreover, no significant difference was observed between baseline (before stimulation) (IFCS before stimulation vs. Sham before stimulation: *p* = .462).

## 4 DISCUSSION

The results of this study indicated that IFCS significantly increased SSF, GEV, and VOC. In contrast, VFS, and SSV did not change with IFCS. In the present study, IFCS applied through electrodes attached to the skin near the cricoid cartilage affected the superior laryngeal nerve, resulting in smoother induction of spontaneous swallowing, which might have led to an increase in the number of spontaneous swallows.

Regarding the mechanism by which IFCS induces spontaneous swallowing, a previous study reported that sensory input to the superior laryngeal nerve promotes the triggering of the swallow reflex (Umezaki et al., 2018). Similarly, it was thought that IFCS induced sensory input to the superior laryngeal nerve, which might have led to the induction of the spontaneous swallow reflex in this study. In animal studies, it has been reported that application to the neck stimulates afferent nerves in the pharynx and larynx; additionally, synaptic inputs are transmitted via the nucleus tractus solitarii and the reticular formation (RF) of the medulla oblongata to the central pattern generator (CPG) of swallowing, and stimulation of the CPG leads to a synergistic effect of the sensory input (Umezaki et al., 2018). Another study reported that swallowing‐related neurons in the medulla oblongata were activated by electrical stimulation of the superior laryngeal nerve (Ootani et al., 1995). Moreover, it was reported that electrical stimulation of the superior laryngeal nerve induced swallowing‐related muscle activity in decerebrate cats (Umezaki et al., 1998). In this study, electrical stimulation (IFCS) of the superior laryngeal nerve may have induced spontaneous swallowing as well as sensory stimulation of the superior laryngeal nerve.

The results of this study indicate that the number of spontaneous swallows increased with IFCS. Conversely, the number of voluntary swallows did not demonstrate a significant increase following IFCS. As a rule, spontaneous swallowing occurs without awareness, such as saliva swallowing, whereas voluntary swallowing occurs under conscious conditions such as eating and drinking. It has been reported that the origin of the swallowing trigger differs between spontaneous and voluntary swallowing. Spontaneous swallowing is induced under the control of the swallowing CPG in the medulla through the transmission of the cortical network, whereas voluntary swallowing requires the transmission of intraoral substances (such as food and saliva) and voluntary motor commands to the tongue and submucosal muscle groups for its initiation (Ertekin, 2011). In addition, spontaneous and voluntary swallowing can be differentiated in terms of the state of consciousness, arousal level, and condition of swallowing (Ertekin, 2011). It has been reported that the number of repetitive salivary swallows is increased by IFCS. However, the details of this process remain unclear. Furthermore, it is also reported that IFCS enhances the swallowing reflex in voluntary swallows, since IFCS possibly leads to stable and continuous stimulation of the peripheral nerves, thereby facilitating voluntary swallows (Kitada et al., 2010). However, the results of this study did not show a significant effect of IFCS on the number of repetitive saliva swallows measured for 30 s based on the common law of RSST. One possible explanation for this result is that the electrical stimulation intensity, although lower than the sensory threshold for the superior laryngeal nerve, may still promote spontaneous swallowing through afferent stimulation. However, it may not be sufficient to impact the excitatory input of higher brain centers that are involved in voluntary swallowing. Another possible reason for this result might be that the measurement time of VSF was only 30 s. In a previous study, Kitada et al. measured the number of voluntary swallows for 1–2 min. The participants in this study were healthy young adults, and the RSST might have had little effect on them in terms of a delayed swallowing reflex due to fatigue of swallowing‐related muscles or difficulty in inducing the swallowing reflex due to decreased salivary volume (Hiramatsu et al., 2015). As such, the participants might be able to perform an increased number of RSSTs. In addition, there was no significant difference between the results of IFCS and the Sham.

The results of this study indicated that GEV and VOC were significantly increased by IFCS. The direct effect of the superior laryngeal nerve on masticatory function has not yet been clarified. Furthermore, the results also suggest that the increase in GEV was related to an increase in the number of chews. According to a previous study, the rhythmic movements of the jaw, tongue, and hyoid (such as during mastication) are determined in the cortical masticatory area (Liu et al., 1993). Moreover, it has been reported that the masticatory CPG is involved in the RF of the medulla oblongata (Sasamoto, 2000). This study also indicates that the input from the superior laryngeal nerve may have affected not only the swallowing neuronal network but also the neuronal network of mastication, which might have increased the number of chews. A previous study reported that masticatory movements evoked by cortical stimulation inhibited laryngeal sensory nerve‐induced swallowing reflex in rats (Tsujimura et al., 2012). According to the results of their study, the swallowing reflex evoked by IFCS to the superior laryngeal nerve inhibited masticatory function, which differs from masticatory movements evoked by cortical stimulation in rats. These results might suggest that mastication suppresses the initiation of swallowing to adjust the initiation of swallowing according to bolus transfer into the pharynx, whereas pharyngeal swallowing does not suppress mastication to prepare next swallowing. However, previous studies and the results of this study suggest a significant relationship between the neuronal network of swallowing and mastication, and further studies are necessary to clarify the details of this relationship between swallowing and mastication. In the present study, we did not measure muscle activity using surface electromyography, nor did we evaluate the activity of the masticatory or supralaryngeal muscles. Thus, it is necessary to evaluate the effect of IFCS on muscle‐related mastication and swallowing to clarify the details of the effects.

In this study, the salivary flow was measured to investigate the changing in the swallowing frequency due to saliva secretion caused by IFCS. The results of this study indicated that no significant effect of the IFCS on salivary flow was observed. A previous study on electrical stimulation reported that electrical stimulation of the submucosal area affects salivary flow (Koike et al., 2020). In that study, the submental region was stimulated, and the target of stimulation was the suprahyoid muscle group. Therefore, it was thought that electrical stimulation of the submental region might have a direct effect on salivary flow or the muscle surrounding the salivary gland. However, no nerves or muscles affected the salivary glands in the region around which the electrode was attached in the present study. Therefore, our findings may not indicate any significant changes in salivary flow. In addition, although the target region in the previous study was different from that in this study, it has been reported that IFCS applied in the submandibular region increases salivary volume in individuals with xerostomia. However, similar to this study, no significant change in salivary volume was observed in healthy participants in that study (Hasegawa et al., 2016). These results suggest that IFCS has little or no effect on the salivary flow in healthy participants, regardless of the region of stimulation.

According to the results of this study, salivary flow did not change significantly, but the number of chews increased. Mastication has been reported to increase salivary flow. It is thought that the activity of the hypothalamus, amygdala, and cerebral cortex, as a result of mastication and the transmission of sensory information from the oral cavity to these higher regions, increases salivary gland activity and promotes salivary flow (Proctor, 2016). One possible reason for the lack of increase in the salivary flow in this study might be that salivary flow was only measured using the Saxon test, which measured the saliva produced during stimulation. The Saxon test indicates that sufficient salivary flow has already been gathered, and the application of IFCS may not indicate increased salivary flow. In addition, salivary flow produced at rest was not investigated in this study. The saliva at rest and during stimulation is produced by submandibular and parotid salivary glands, respectively. The differences between the properties and composition of saliva between the two types of saliva have been previously reported (Proctor & Carpenter, 2014). Saliva produced during stimulation was promoted by the stimulation transmitted to afferent nerves when sensory receptors such as taste, touch, and temperature were activated. These nerves form a reflex arch with lower centers such as the medulla oblongata and spinal cord as reflex centers and postnodal fibers such as sympathetic and parasympathetic nerves distributed within the fibrous tissue of salivary glands to stimulate salivary flow (Yoshida & Nishikawa, 1995). The Saxon test, which only increases salivary flow through mastication, suggests that IFCS does not have a significant effect on salivary flow directly. However, it was thought that the increasing number of chews caused by IFCS could promote salivary flow. Therefore, it is necessary to evaluate the components of saliva during stimulation and the resting salivary volume to investigate the effect of IFCS on salivary flow.

This study has several limitations. First, because healthy adults were included in the present study, no significant differences were found in the number of VSFs due to the high values. Therefore, elderly people and patients with dysphagia should be considered as participants in future studies. Second, in this study, IFCS and sham stimulation was performed in random order. Third, the participants remembered the properties of the gummies used in this study in the first measurement, which might have influenced the results regarding mastication in the second measurement. Therefore, it is necessary to evaluate them using a variety of shapes. Moreover, the results of this study suggest that IFCS may influence masticatory function. However, various factors influence masticatory efficiency, including occlusal contact conditions and bite forces. Therefore, a more detailed assessment of these other factors is necessary to examine the impact of IFCS. Fourth, salivary secretion during stimulation should also be investigated, in addition to saliva at rest. Finally, IFCS and Sham were blinded by using a perceptual threshold just below the discomfort sensation to eliminate the participants’ preconceived notions of what they were receiving electrical stimulation in this study. However, compared to NMES, IFCS is an electrical stimulus with the advantage that it can be used with less muscle contraction and pain even when the stimulus intensity is increased (Oku, 2021). Therefore, it has the potential that increasing stimulus intensity may affect the results. Thus, it is necessary to use IFCS with changing stimulation intensity to verify the maximum effect. As this was an exploratory study and multiplicity has not been investigated, it is considered an issue for future study.

## 5 CONCLUSION

The results of this study indicated that IFCS significantly increased SSF, GEV, and VOC. These findings suggest that IFCS of the superior laryngeal nerve, a swallowing‐related nerve, might affect masticatory and swallowing function.

## AUTHOR CONTRIBUTIONS

**Yoshiki Iizumi:** Writing—original draft preparation; data curation. **Yoshiaki Ihara:** Conceptualization; formal analysis; writing—original draft preparation. **Joji Koike:** Formal analysis; funding acquisition. **Koji Takahashi:** Supervision. All authors reviewed the manuscript.

## CONFLICT OF INTEREST STATEMENT

The authors declare no conflict of interest.

## ETHICS STATEMENT

The study protocol of this study was approved by the Ethics Committee of Showa University (Approval no. DH20200182). The study was conducted in accordance with all the provisions of the World Medical Association Declaration of Helsinki (version 2002). All methods, involving human participants, were performed in line with the relevant guidelines and regulations of the Ethics Committee of Showa University. Before participating in this study, all patients received both oral and written informed consent and signed an approved written informed consent form.

## Data Availability

The datasets generated and analyzed during the current study are available from the corresponding author upon reasonable request.
